# Exosome microRNA signatures in patients with complex regional pain syndrome undergoing plasma exchange

**DOI:** 10.1186/s12967-019-1833-3

**Published:** 2019-03-14

**Authors:** Sujay Ramanathan, Sabrina R. Douglas, Guillermo M. Alexander, Botros B. Shenoda, James E. Barrett, Enrique Aradillas, Ahmet Sacan, Seena K. Ajit

**Affiliations:** 1 Pharmacology & Physiology, Drexel University College of Medicine, 245 North 15th Street, Mail Stop 488, Philadelphia, PA 19102 USA; 2 Neurology, Drexel University College of Medicine, 245 North 15th Street, Philadelphia, PA 19102 USA; 3 School of Biomedical Engineering, Science & Health Systems, Drexel University, 3141 Chestnut Street, Philadelphia, PA 19104 USA; 4 Present Address: Vincera Institute, Philadelphia, PA 19112 USA

**Keywords:** Plasma exchange, Exosomes, miRNA, Inflammation, Biomarker

## Abstract

**Background:**

Therapeutic plasma exchange (PE) or plasmapheresis is an extracorporeal procedure employed to treat immunological disorders. Exosomes, nanosized vesicles of endosomal origin, mediate intercellular communication by transferring cargo proteins and nucleic acids and regulate many pathophysiological processes. Exosomal miRNAs are potential biomarkers due to their stability and dysregulation in diseases including complex regional pain syndrome (CRPS), a chronic pain disorder with persistent inflammation. A previous study showed that a subset of CRPS patients responded to PE.

**Methods:**

As a proof-of-concept, we investigated the PE-induced exosomal miRNA changes in six CRPS patients. Plasma cytokine levels were measured by HPLC and correlated with miRNA expression. Luciferase assay following co-transfection of HEK293 cells with target 3′UTR constructs and miRNA mimics was used to evaluate miRNA mediated gene regulation of target mRNA. Transient transfection of THP-1 cells with miRNA mimics followed by estimation of target gene and protein expression was used to validate the findings.

**Results:**

Comparison of miRNAs in exosomes from the serum of three responders and three poor-responders showed that 17 miRNAs differed significantly before and after therapy. Of these, poor responders had lower exosomal hsa-miR-338-5p. We show that miR-338-5p can bind to the interleukin 6 (IL-6) 3′ untranslated region and can regulate IL-6 mRNA and protein levels in vitro. PE resulted in a significant reduction of IL-6 in CRPS patients.

**Conclusions:**

We propose that lower pretreatment levels of miR-338-5p in poor responders are linked to IL-6 levels and inflammation in CRPS. Our data suggests the feasibility of exploring exosomal miRNAs as a strategy in patient stratification for maximizing therapeutic outcome of PE.

**Electronic supplementary material:**

The online version of this article (10.1186/s12967-019-1833-3) contains supplementary material, which is available to authorized users.

## Background

Therapeutic plasmapheresis or plasma exchange (PE) is an extracorporeal blood purification technique designed to remove large-molecular-weight substances [1]. For PE to be a rational choice as a blood purification technique, at least one of the following conditions should be met. The substance to be removed is sufficiently large (≥ 15 kDa), has a comparatively prolonged half-life so that its removal provides a therapeutically beneficial period of reduced serum concentration, or the rapid removal of acutely toxic substance resistant to conventional therapy is clinically indicated [1]. PE is a widely used therapeutic procedure in many immunologic renal and neurological disorders, enabling the removal of pathogenic antibodies and circulating immune complexes that can cause inflammation. Often used in combination with immunosuppressive therapies, PE has led to a steady increase in survival rates over the last 40 years in these diseases with a poor prognosis without treatment [2].

Complex regional pain syndrome (CRPS) is a chronic neuropathic pain disorder and the patient population is heterogeneous with symptoms encompassing sensory, motor and autonomic dysfunction. This disabling disease is characterized by allodynia (pain from a non-painful stimulus), hyperalgesia (heightened sensitivity to pain), changes in skin color, temperature, edema, alterations in hair, skin or nail, dystonia, and tremors [3, 4]. The pathophysiological mechanisms of CRPS are not fully understood, although a moderate association has been observed with autoantibodies against adrenoceptors [5]. PE can remove proinflammatory cytokines, immune complexes and autoantibodies to adrenoceptors, and thus relieve localized and systemic symptoms [6–9]. A recent study reported that PE was effective on a subset of CRPS patients with reduced epidermal nerve fiber density count, consistent with a small fiber neuropathy, with 30 out of 33 (91%) patients reporting pain relief [10].

In addition to high molecular weight entities, plasma also contains extracellular vesicles including the 30–150 nm exosomes. These circulating exosomes are secreted by a variety of cell types and transport various cargo molecules including miRNA, long noncoding RNA, mRNA, lipids, DNA and proteins. The release and uptake of exosomes play an important role in intercellular communication [11]. The content of exosomes is regulated under homeostasis, and often a dysregulation precedes or ensues pathology [12, 13]. Distinct expression patterns of circulating miRNAs have been associated with a number of diseases [14]. Widely recognized for their role as fine tuners of gene expression, miRNAs that mediate posttranscriptional regulation influence virtually all aspects of cellular processes and are also transported via exosomes [11, 15, 16]. These small noncoding RNAs regulate gene expression by binding predominantly to the 3′ untranslated region (3′UTR) of mRNAs by 6- to 8-basepair seed sequence complementarity. Upon binding, miRNAs can induce mRNA degradation or translational repression and thus negatively regulate the expression of target genes [15, 16].

A recent study investigated whether the removal or reduction of circulating miRNAs with plasma exchange improved pathologies caused by miRNAs [17]. The study evaluated miRNAs in three patients with systemic lupus erythematosus undergoing plasmapheresis and showed that a large number of circulating miRNAs in plasma were separated with apheresis [17]. Our previous study has shown that a larger number of circulating miRNAs are differentially expressed (or packaged) into exosomes in CRPS patients compared to exosomes from control subjects [18]. To the best of our knowledge, there are no studies to date investigating exosome composition in serum before and after PE. Thus, we sought to determine the feasibility of using circulating miRNAs transported within exosomes in serum samples of CRPS patients before and after PE as a potential molecular signature or biomarker, by performing a proof-of-concept study in three patients who responded and three patients who did not respond to PE.

## Methods

### Standard protocol approvals, registrations, and patient consents

This is a retrospective case series study of CRPS patients seen at the Drexel University College of Medicine pain clinic that met the Budapest consensus criteria for CRPS and received PE as treatment for their illness between September 2012 and June 2014. All participants agreed to provide blood samples for this study after giving informed consent as approved by the institutional review board.

### Plasma exchange and patient evaluation

The patients were asked to provide blood samples before and after PE. Patients with autoimmune or immunodeficiency conditions were excluded. Patient records were reviewed from which data regarding demographics, CRPS signs and symptoms, duration of illness and response to PE were obtained. PE was performed in patients with refractory CRPS over 2 weeks at the Hahnemann University Hospital, Philadelphia. All patients had full cardiac and neuropsychological clearance before PE. Patients were asked to rate their overall pain before, during, and after the apheresis using a 11-point numerical rating scale (NRS) from 0 (no pain) to 10 (the worst pain imaginable).

### Characterization of exosomes by nanoparticle tracking analysis, electron microscopy and western blot analysis

One milliliter of serum from CRPS patients was diluted in equal volume of PBS and centrifuged at 2000×*g* for 30 min at 4 °C. The supernatant was diluted to final volume of 24 ml in 1× PBS and centrifuged at 12,000×*g* for 45 min at 4 °C. The supernatant was filtered through a 0.22 µ filter and centrifuged at 110,000×*g* for 70 min at 4 °C. The exosome pellet obtained was washed in 25 ml 1× PBS without ions and centrifuged at 110,000×*g* for 70 min at 4 °C. The exosome pellet was resuspended in 100 µl of PBS for use in nanoparticle tracking analysis, electron microscopy and protein estimation.

### Nanoparticle tracking analysis

Exosomes in PBS were analyzed for size and concentration using the NanoSight NS300 according to the manufacturer’s protocol (Malvern Instruments, MA, USA). Samples were diluted to ~ 10^7^–10^9^ particles/ml and continuously injected with a syringe pump and three videos (30 s each) were captured for particle analysis. Nanoparticle tracking analysis was performed using NTA 3.2 software.

### Electron microscopy

Ten microliters of PBS resuspended exosomes were coated on Ni-formvar grids and incubated for 20 min at RT. The grids were washed on 50 µl drops of 0.1 M Sorensen’s phosphate buffer (pH 7.2) for 5 s each for a total of five times. The grids were blot dried perpendicularly on whatman #1 filter paper. Negative staining and embedding were performed by incubating the grids on 0.5% uranyl acetate (in a 0.2% methyl cellulose solution) for 10 min at 4 °C. The excess solution was blotted on a Whatman paper, air dried and imaged in a JEOL Transmission Electron Microscope (JEM 1230). Alternatively, the exosomes were immunolabelled for the exosome marker CD81 and crosslinked with 1% glutaraldehyde, and probed with 6 nm gold secondary antibody, followed by negative staining and embedding.

### Western blotting

Protein concentration was estimated using a DC Protein assay (Bio-Rad Laboratories, CA, USA); 5 µg of exosomes isolated from CRPS serum were resolved on a reducing 12% SDS-PAGE, transferred to PVDF membrane and blocked in Odyssey Blocking buffer (927-50100, LI-COR Biosciences) for 2 h. The membrane was incubated in rabbit anti-CD63 antibody (ab68418, Abcam) overnight at 4 °C, washed thrice in TBST, 10 min each, and incubated in goat anti-rabbit 680RD IgG (925-68071, LI-COR Biosciences) for 45 min at RT, washed thrice in TBST for 10 min each, and imaged on an Odyssey Fc imaging system.

### Exosome miRNA profiling

RNA was isolated from exosomes using a miRvana miRNA isolation kit (Life technologies) following the manufacturer’s protocol. Taqman low-density array microfluidic cards version A and B (Applied Biosystems, Foster City, CA) were used to profile miRNAs in 100 ng of total RNA as previously described [19]. miRNA species with CT values 35 and higher were treated as undetected. Fold change was calculated from raw CT values using the 2^−ΔΔCT^ method [20]. The mean CT values of the 10 miRNAs with the lowest standard deviations across all samples were used as the endogenous control in the calculation of ΔCT. Statistical significance of differences in ΔCT values was calculated by a two-tailed paired t-test for comparison of pre- and post-PE samples and by a two-tailed independent t-test for comparison of other experimental groups. A p-value threshold of 0.05 and a fold-change of 2 were used to select significant differentially expressed miRNAs between experimental groups.

### Cell culture

HEK293 cells obtained from the American Type Culture Collection (ATCC) was maintained in Dulbecco’s Modified Eagle’s Medium (DMEM) supplemented with 10% fetal bovine serum at 37 °C in 5% CO_2_. Human monocytic THP-1 cells (TIB-202, ATCC) cells were maintained in RPMI-1640 medium containing 10% fetal bovine serum (FBS).

### Luciferase reporter assay

The 3′UTR luciferase reporter constructs for IL-6 (NM_000600.2) was purchased from GeneCopoeia. The miRNA mimics for human miR-338-5p (MC12825), anti-miR-338 (MH12825) and scrambled negative control (4464058) was purchased from Life technologies. HEK293 cells were co-transfected with mir-338-5p, anti-miR-338 or miRNA scrambled control and luciferase reporter plasmid containing the 3′UTR of human IL-6 using Lipofectamine LTX (Life Technologies, Carlsbad, CA) for 48 h. The Luc-Pair miR Luciferase assay kit (GeneCopoeia) was used to measure firefly and Renilla luciferase activity according to the manufacturer’s instruction. Firefly luciferase measurements normalized to Renilla luciferase was used as a transfection control. The data expressed as percentage of control is the average of three independent experiments.

### miR-338-5p overexpression in THP-1 cells

Transfections were performed following the manufacturer’s protocol for RNAiMax transfection reagent using either miR-338-5p or negative control with the following modifications. For each well of a 6-well plate, 7.5 µl RNAiMax reagent was diluted in 150 µl of serum-free media, and 30 pmol of miR-338-5p or control mimic was diluted in 150 µl of serum-free media individually. The dilutions were combined and incubated at room temperature for 15 min. This transfection complex (300 µl) was added to 0.5 × 10^6^ cells/well in 1.7 ml serum containing media in 6-well plates and incubated for 6 h at 37 °C, after which the media was changed. After 24 h, cells were treated with 1 µg/ml lipopolysaccharide (LPS) in complete culture media for 6 h. Exosome deplete media was used in all experiments. Cells were collected by centrifugation at 135×*g* for 5 min at 4 °C and the conditioned media was stored at 4 °C. The cell pellet was washed with 1× PBS and resuspended in either RNA lysis buffer (mirVana kit; Life Technologies) containing 0.5 U/µl RNAsin Plus (Promega; Madison, WI) for RNA isolation or 1× radioimmunoprecipitation assay (RIPA) buffer containing protease inhibitor cocktail (Thermo Scientific; Waltham, MA) for protein analysis.

### cDNA synthesis and qPCR for mRNAs

mRNA was isolated using the miRVana kit (Life technologies). The Maxima cDNA synthesis kit (Thermo Scientific) was used to generate cDNA and 2 µl cDNA was used for Taqman based quantitative real time mRNA analysis containing 10 µl Taqman Fast Universal polymerase chain reaction (PCR) master mix (2×) no AmpErase UNG (Life Technologies), 1 µl Taqman primer–probe (20×), in up to 20 µl nuclease-free water. GAPDH was used as the normalizer and one-way ANOVA was used to perform statistical analysis. Assay IDs were as follows: Hs00985639_m1 [IL-6], 4325792 (GAPDH) (Applied Biosystems, Carlsbad, CA).

### Enzyme-linked immunosorbent assay (ELISA) for IL-6 in cell culture media

Supernatants collected after miR-338-5p or control miRNA transfections in THP-1 cells were used to perform ELISA for secreted IL-6 using the human IL-6 quantikine ELISA kit (D6050) according to the manufacturer’s protocol (R&D Systems; Minneapolis, MN).

### Cytokine measurement in patient plasma

For plasma isolation, blood was collected into EDTA-coated (purple top) vacutainers. The plasma was separated by centrifugation (3000×*g* for 15 min at 4 °C), split into 250 µl aliquots and stored at − 70 °C until assayed. The MILLIPLEX MAP Human High Sensitivity T Cell Panel, HSTCMAG-28SK (Millipore, Billerica, MA) was used to determine plasma levels of 14 cytokines. The minimum detectable concentration for IL-6 and TNF-α was 0.11 and 0.16 pg/ml respectively. All assays were performed in duplicate according to the manufacturers’ instructions. Assay results were determined on a Luminex-200 (Luminex, Austin, TX).

### Statistical analysis

Data are presented as mean ± the standard error of the mean from three or more independent experiments. Student *t*-test was used for determining the statistical significance. Treatment effects were analyzed with a one-way analysis of variance (ANOVA). Pairwise comparisons between means were tested using the post hoc Dunnet method. Error probabilities of *p* < 0.05 were considered statistically significant. Paired t-test with Bonferroni correction for multiple assays was used for analysis of the cytokine panel. Error probabilities of *p* < 0.00357 were considered statistically significant.

## Results

### Isolation and characterization of exosomes

**Fig. 1:**
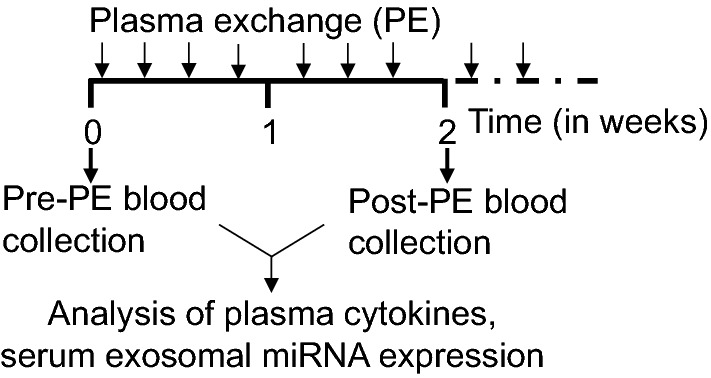
Schematic of plasma exchange (PE) protocol and sample collection for downstream analysis. Blood was collected from CRPS patients before PE and following a series of five to seven more PE distributed over 2 weeks. Plasma was used to assay the cytokines, while serum was used for exosome isolation and downstream miRNA profiling

**Fig. 2:**
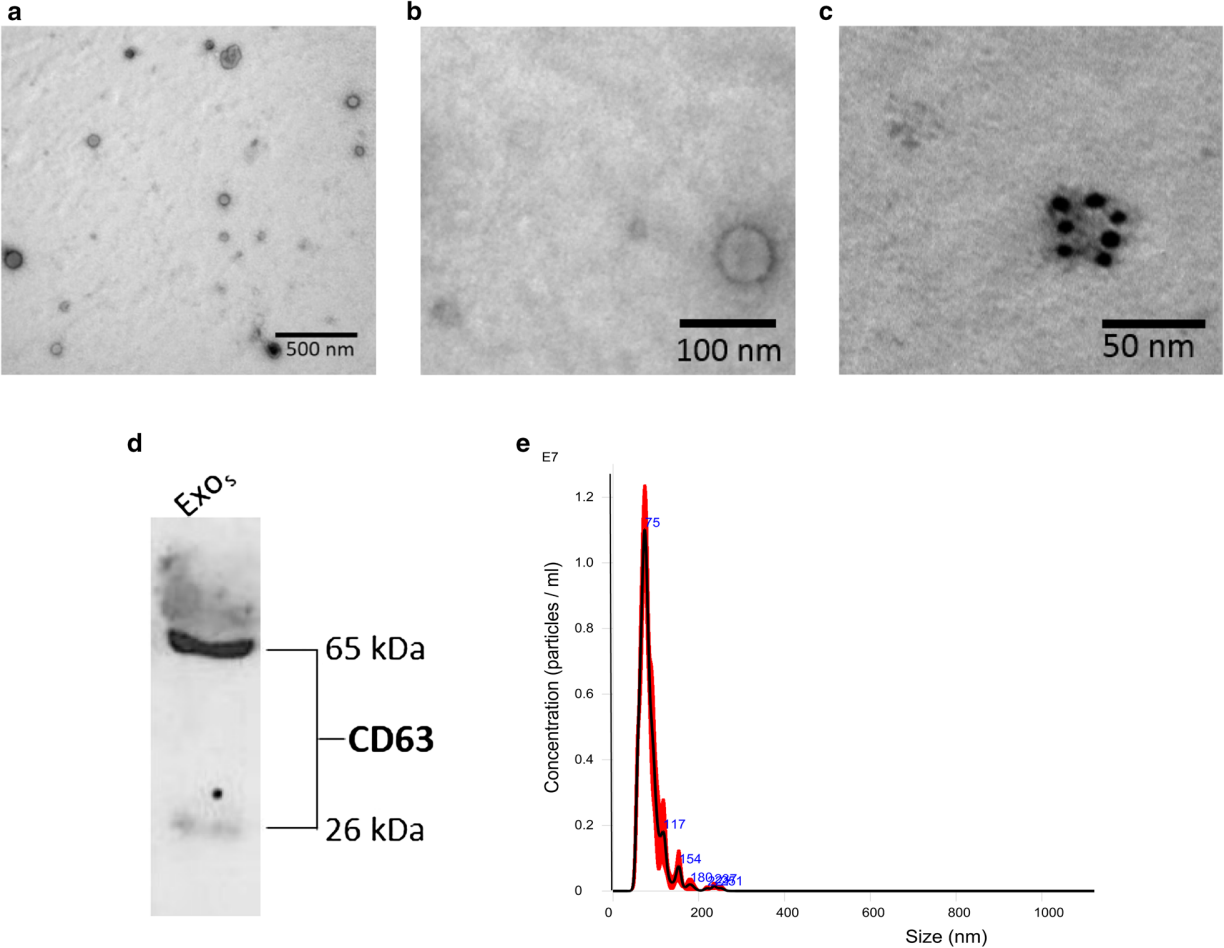
Characterization of exosomes purified from the serum of CRPS patients. Exosomes were isolated from the serum of CRPS patients using differential ultracentrifugation. The exosomes were observed by TEM under a Toshiba H8000 or JEM1230 microscope. Exosomes were fixed and negatively stained with uranyl acetate for morphology (**a**, **b**) or immunolabelled for the exosome marker CD81 and probed with 6 nm gold secondary antibody, following negative staining (**c**). Exosome proteins isolated from PE patient serum (Exo_s_) were resolved on a reducing 12% SDS-PAGE and western blotting was used to confirm the presence of exosome marker protein CD63 (**d**). Nanoparticle tracking analysis of serum exosomes indicated particles with a mean diameter of 85.7 ± 0.9 nm and a particle concentration of 3.94 × 10^11^ ± 1.6 × 10^10^ particles/ml (**e**)

Exosomes were isolated from the serum obtained from six CRPS patients, grouped retrospectively as three responders and three non-responders both before and after PE (Fig. 1, Table 1). The exosomes displayed a size below 100 nm under a transmission electron microscope (TEM) (Fig. 2a, b). Exosomes are characterized by the presence or enrichment of endosome-derived membrane proteins such as tetraspanins (CD9.CD63, CD81), flotillins, Alix and Tsg101. The presence of such characteristic exosomal proteins was also assessed using immunogold labeling technique for CD81 by TEM (Fig. 2c) and western blotting for CD63 (Fig. 2d). Nanoparticle tracking analysis of serum exosomes indicated particles with a mean diameter of 85.7 ± 0.9 nm and a concentration of 3.94 × 10^11^ ± 1.6 × 10^10^ particles/ml (Fig. 2e). The metrics of exosome isolation, purity and characterization was submitted on EV track [21] and the EV-metric score is 44%.

**Table 1:** Pain assessment of CRPS patients before and after PE

ID	Age	Sex	Duration of CRPS (years)	Pre PE NRS	Post PE NRS	HT (cm)	WT (kg)	BMI	Response	Comorbidities
P1	54	F	4	7	3	170	100.9	34.9	Good	Hypothyroidism, B12 deficiency, Iron deficiency, GERD, Seizure,
P2	40	M	11	8	4	195	126	33.1	Good	No past medical history other than CRPS
P3	57	M	> 6	8	2	165	120	44.1	Good	Asthma, DM, OSA, Hypercholestrolemia, Mitochondrial disease
P4	57	F	> 6	8	7	163	51.63	19.4	Poor	Fibromyalgia, Anemia
P5	28	F	7	7	7	160	81.1	31.7	Poor	Grave’s disease, Polycystic ovary syndrome, Migraine, Von Willebrand disease
P6	26	F	> 6	8	7	163	52.27	19.7	Poor	Stiff man syndrome, Seizure disorder

Pain assessment was conducted on CRPS patients before and 2 weeks following PE, using a numerical rating scale (NRS). CRPS patients with a difference in the NRS pain scores (pre PE-post PE) greater than three were considered as responders, while those less than three were classified as poor responders

### Differentially expressed miRNAs between responders and poor responders of PE

**Fig. 3:**
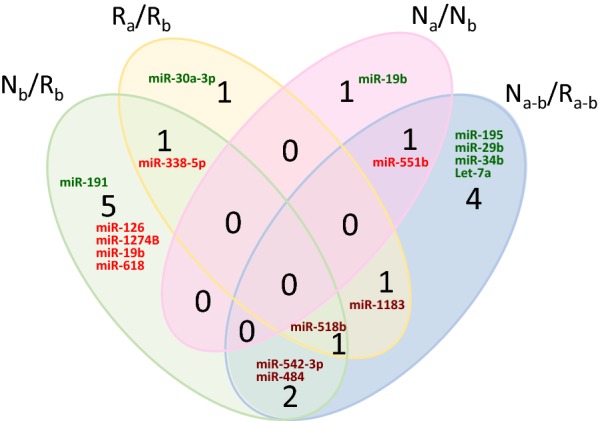
Exosomal miRNA expression in CRPS patients undergoing plasma exchange. A venn diagram showing relative expression of various miRNAs evaluated from serum exosomes of six CRPS patients before PE and 2 weeks following it. Comparison of miRNA levels are presented in responders (R) and poor responders (N) before (b) and after (a) PE. Upregulated miRNAs are shown in green and downregulated miRNAs in red, while miRNAs that are common between the compared groups but differing in directionality are in dark red. The significance was determined using student t-test (p < 0.05)

Exosomal miRNAs isolated from six CRPS patients, before and after PE, were used to profile a total of 754 miRNAs. The mean CT values of the 10 miRNAs with the lowest standard deviations across all samples were used as the endogenous control in the calculation of ΔCT excluding miRNAs with a CT value of > 35 in any sample. Statistical significance of differences in ΔCT values was calculated by a two-tailed paired t-test for comparison of pre- and post-PE samples and by a two-tailed unpaired t-test for comparison of other experimental groups. A p-value threshold of 0.05 and a fold-change of 2 were used to select significantly differentially expressed miRNAs between experimental groups (Table 2, Fig. 3, Additional file 1: Table S1). The comparison between two groups is depicted as log2 fold change values. A positive number in the fold change for nb/rb, it means nb is higher than rb. A negative fold change number for nb/rb comparison means nb is lower. To investigate differential miRNA expression between responders and non-responders prior to PE, we analyzed the miRNAs from the pretreatment groups (column 2 of Table 2, Fig. 3). Of the nine miRNAs that were significantly different between responders and non-responders prior to treatment, two were upregulated and seven downregulated in non-responders relative to responders. This suggests that the exosomal molecular signature differed even prior to PE and could be explored further for biomarker potential in predicting treatment response. To determine which miRNAs changed in responders after treatment, miRNA profiles before and after treatment were compared (Table 2, column 3). Four miRNAs changed significantly in responders after treatment with two miRNAs upregulated and two downregulated (Fig. 3). Two miRNAs were altered after PE in non-responders; however, these were different from the miRNA changes observed in responders. To determine differences in miRNA alterations by PE between responders and non-responders, we compared the changes in miRNA levels of each patient. Results from this meta-analysis (Table 2, column 5) shows nine miRNAs that have different degrees of change in responders and poor responders. For example, hsa-let-7a is downregulated in both responders and non-responders, but the degree of change in responders is 4.5-fold greater than in non-responders.

**Table 2:**
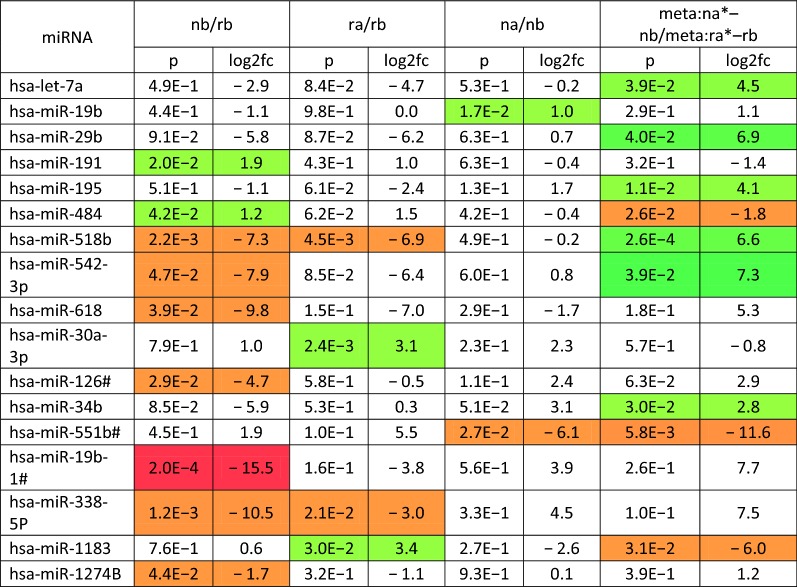
Relative expression of exosomal miRNAs in CRPS patients undergoing plasma exchange (PE)

Relative expression of exosomal miRNAs in six CRPS patients pre and post-PE were determined using a Taqman low density array. The significance was determined using two-tailed paired t-test for comparison of pre- and post-plasma exchange samples and by a two-tailed independent t-test for comparison of other experimental groups. A p-value threshold of 0.05 and a fold-change of 2 were used to select significantly differentially expressed miRNAs between experimental groups

Log2fc: Log 2 fold change, orange red: downregulation, green: upregulation, Rb: responders before PE, Nb: non-responders before PE, Ra: responders after PE, Na: non-responders after PE, Na-Nb/Ra-Rb (miRNA changes in non-responders over responders as a result of PE)

In order to ascertain the potential function of these miRNAs, we employed a combination of bioinformatic algorithms Targetscan and miRDB that predict miRNA targets based on the ability of the miRNA sequence to undergo specific base-pairing within the putative 3′UTR in the mRNA target [22, 23]. Among the pro-inflammatory targets, IL-6 was among the top candidates for both miR-19a/b and miR-338-5p [22, 24]. The regulatory role of miR-19 on IL-6 has been demonstrated and shown to be indirectly mediated by the binding of miR-19a and miR-19b to Toll-like receptor 2 [25]. The role of miR-338-5p in inflammation or CRPS however has not yet been explored. This led us to investigate the biological role of miR-338-5p in regulating inflammatory responses.

### Confirmation of miR-338-5p binding to the 3′UTR of predicted targets

**Fig. 4:**
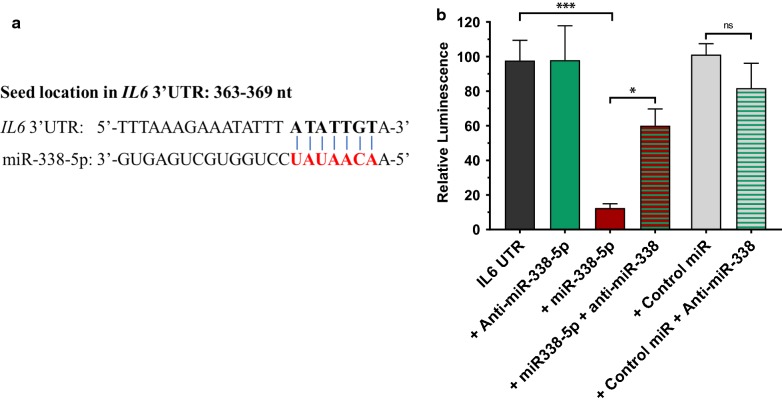
Luciferase assay to determine miR-338-5p binding to the 3′UTR of predicted mRNA targets. **a** The miR-338-5p targeting sequence in the 3′-untranslated region of human IL6 mRNA. **b** Plasmids with the 3′UTR of IL-6 cloned downstream of the luciferase open-reading frame were cotransfected with miR-338-5p, anti-miR-338 or control miRNA mimic in HEK293 cells. Luciferase activity was measured 48 h after transfection, and the data expressed as percentage of luminescence. Firefly luciferase measurements were normalized to Renilla and the average of three independent experiments is shown. Statistically significant difference from control was calculated using Student t-test, *p < 0.05

The predicted interaction of miR-338-5p and IL-6 mRNA (Fig. 4a) was studied using a luciferase reporter assay. The 3′UTR of human IL-6 harboring miR-338-5p binding sites cloned downstream of the luciferase open-reading frame was used to assess miRNA-mRNA binding. HEK293 cells were transiently transfected with plasmids encoding the reporter 3′UTR construct and either miR-338-5p mimic, anti-miR-338 or a miRNA control. Firefly luciferase measurements were normalized to Renilla luciferase as a transfection control. A significant reduction was observed in luciferase activity 48 h after transfection with miR-338-5p, (p < 0.05) confirming the binding of miR-338-5p to the 3′UTR of IL-6 mRNA (Fig. 4b). Anti-miRs are complementary to the target miRNAs and used to study loss of function effects of specific miRNAs. Transfection with anti-miR-338 individually or in combination with control miRNA did not alter relative luminescence but when combined with miR-338-5p, significantly reversed the inhibition indicating downregulation of miRNA activity.

### Transcriptional and translational regulation of IL-6 mRNA by miR-338-5p

**Fig. 5:**
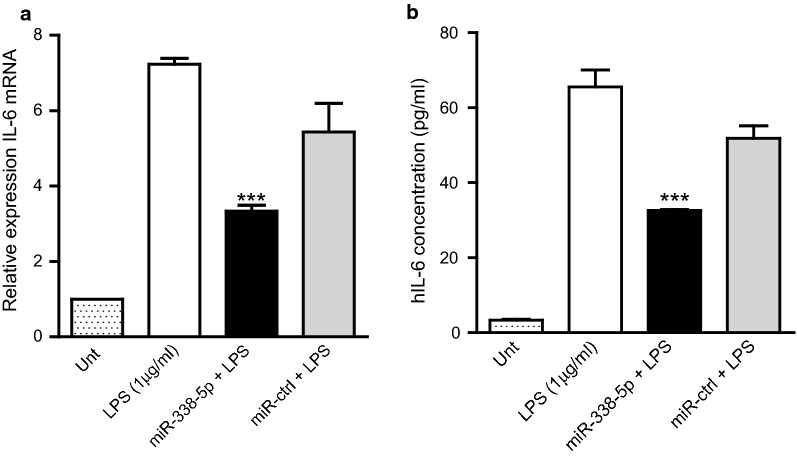
Expression levels of IL-6 mRNA and protein in THP-1 cells transfected with miR-338-5p followed by 6 h of stimulation with LPS. **a** Taqman analysis of endogenous levels of IL-6 after LPS induction, showed that transfection with miR-338-5p reduced IL-6 transcripts compared to control miRNA (miR-ctrl). **b** Overexpression of miR-338-5p decreased the secreted IL-6 content. ELISA using cell culture supernatants of THP-1 cells stimulated with LPS showed lower levels of the proinflammatory mediator secreted by miR-338-5p transfected cells compared to control miRNA transfection. Significance was determined by one-way ANOVA with Dunnet’s post hoc test, ***p < 0.001

We next assessed the ability of miR-338-5p to modulate levels of the pro-inflammatory IL-6 mRNA in vitro. IL-6 mRNA and protein levels are usually very low in normal/unstimulated immune cells. Hence, to assess the observable effects of miR-338-5p on IL-6 mRNA, we stimulated human monocytic leukemia (THP-1) cells with bacterial lipopolysaccharide (LPS). THP-1 cells were transfected with either miR-338-5p mimic or control miRNA for 18 h and then stimulated with LPS (1 µg/ml) for 6 h, following which endogenous levels of target mRNAs were measured by qPCR. There was a significant reduction in IL-6 mRNA upon miR-338-5p transfection compared to control miRNA transfection, suggesting that miR-338-5p regulates IL-6 via mRNA degradation (Fig. 5a). Conditioned media from THP-1 cells transfected with miR-338-5p or control and stimulated with LPS were analyzed by ELISA for IL-6 protein levels. The overexpression of miR-338-5p significantly reduced protein levels of IL-6 secreted into the media (Fig. 5b). Thus, over expression of miR-338-5p resulted in downregulation of both mRNA and protein levels of IL-6.

### Plasma levels of cytokines in CRPS patients

**Fig. 6:**
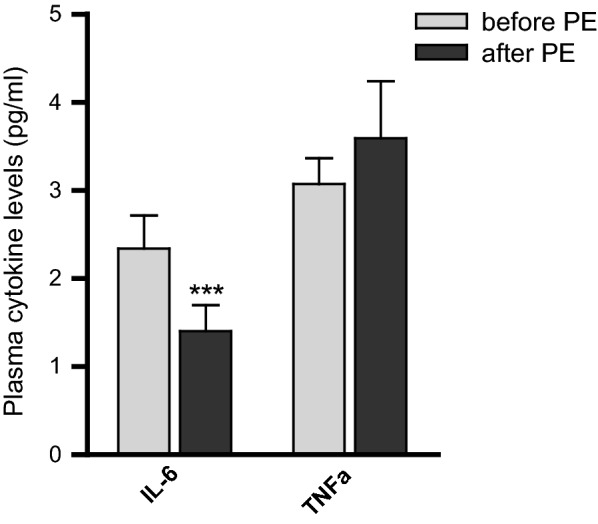
Changes in plasma levels of IL-6 and TNF alpha in CRPS patients following plasma exchange (PE). Plasma levels of IL6 and TNFα were evaluated in plasma from 18 CRPS patients before and 2 weeks following PE. IL-6 was significantly lower following PE but TNFα was not. The significance was determined using student paired t-test (***p < 0.001)

Persistent inflammation is a hallmark of CRPS and an upregulation of proinflammatory cytokines has been demonstrated by multiple approaches in various biofluids including plasma, serum, blister fluid and cerebrospinal fluid [26]. We investigated a panel of 14 cytokines (Additional file 2: Table S2) of which only IL-6 was significantly different following PE. As both miR-19 and miR-338-5p can target IL-6, we investigated IL-6 in a plasma sample repository available from 18 patients. A significant reduction was observed in the plasma levels of IL-6 following PE (n = 18), while TNFα levels did not change (Fig. 6).

## Discussion

Exosomes derived from the endocytic pathway contain biomolecular cargo that is reflective of the physiological state of the cells secreting them [27]. Thus, exosomes provide a snapshot of a molecular signature indicative of the changes resulting from disease or therapeutic intervention. Furthermore, miRNAs transported by exosomes regulate gene expression in recipient cells [28]. Efficacy of PE in immunological, metabolic diseases, and intoxications has been attributed to the removal of humoral factors and autoantibodies [2]. Decreases in T-lymphocyte activation, B lymphocytes and proinflammatory plasma cytokines from PE has also been reported [29]. CRPS is notoriously difficult to treat [3, 4]; however there are few reports suggesting that PE can manage symptoms, presumably by removing the patient’s autoantibodies [6, 8, 10, 30]. One study evaluating the efficacy of PE in refractory CRPS patients with a clinical presentation of a small fiber neuropathy suggested that patients with the greatest loss of small fibers and temperature sensory deficits are more likely to benefit from PE [10]. Using a multi-pronged approach of immune-modulating agents and IVIG, in addition to a weekly PE regime, 91% efficacy was observed in this previously treatment resistant patient group [10]. In these patients, pain reduction was either maintained with immune modulating therapies, or the pain gradually returned to pre-treatment levels. While successful in the aforementioned study, the therapeutic benefit from PE is variable. Moreover, there is no universally efficacious treatment for CRPS, and the mechanism by which pain evolves in CRPS or how treatments such as PE reduce pain in CRPS are unknown. All these factors necessitate the identification of a prognostic biomarker or a molecular signature to predict therapeutic efficacy of PE. We investigated the feasibility of studying exosomal miRNAs in serum of CRPS patients before and after PE and the regulatory role of one of the differentially expressed circulating miRNAs.

The purity and integrity of exosomes isolated conform to the guidelines put forth by the International Society for Extracellular Vesicles [21, 31] and the EV-METRIC score reported is based on the experimental parameters used. Differential expression of several miRNAs was observed in exosomes derived from responders and non-responders to PE. Since this was a retrospective study and the therapeutic outcome is known, we asked if the exosomal miRNA signature differed even prior to PE by comparing the pretreatment groups of responders and non-responders. Our analysis showed differential expression of nine miRNAs between responders and non-responders prior to treatment, suggesting two heterogeneous group of patients. This was further affirmed by the observation that PE-induced miRNA changes also differed between responders and non-responders when pre and post PE samples were compared. Both the above mentioned observations are consistent with a previous study investigating circulating miRNAs in whole blood of CRPS patients receiving ketamine therapy [32].

Amongst the differentially expressed miRNAs, miR-338-5p showed a downregulation in poor responders compared to responders. Though the mechanisms underlying the development of pain are not well understood, inflammation is known to play a crucial role in CRPS. CRPS patients have significantly increased proinflammatory cytokines compared to controls [26]. Based on the bioinformatics prediction that miR-338-5p can bind the 3′UTR of *Il6* mRNA, we sought to confirm the interaction between them. The seed sequence of miR-338-5p is ACAAUAU. In vitro validation studies confirmed the interaction between miR-338-5p and IL-6. IL-6 levels in plasma was higher in responders before PE and considerably decreased following PE. Thus, removal of IL-6 due to PE can contribute directly to reduction in inflammation. Our studies separating responders and poor responders suggest that higher IL-6 levels (before PE) in responders are linked to miR-338-5p levels. In other words, higher IL-6 levels in responders may mediate intercellular signaling via exosomal miR-338-5p to resolve inflammation, and when the proinflammatory IL-6 is removed from circulation by PE, exosomal miR-338-5p is also significantly lowered (Table 2, Additional file 2: Table S2). Poor responders did not exhibit a notable reduction in plasma IL-6 following PE, and hence there was no significant shift in the exosomal miR-338-5p levels. Taken together, these data suggest that determining the expression levels of miRNAs transported by exosomes in combination with cytokine analysis may be a feasible approach for patient stratification and may help predict therapeutic efficacy of PE.

The limitation of our study is the small number of patients (n = 6), and its retrospective nature including non-randomization. Another aspect to consider are the comorbidities associated with CRPS, how they contribute to and impact inflammation and pain. We have previously observed that CRPS patients responding poorly to ketamine had a lower body mass index (BMI) relative to responders [33]. Here we observed that all three responders have high BMI, and two out of three poor responders have lower BMI. Additional studies representing a larger patient cohort and analysis of an array of immune markers are warranted to further investigate these preliminary observations.

## Conclusions

This study provides the first evidence of differential regulation of exosomal miRNAs in CRPS patients undergoing PE. Our proof-of-concept study indicates that investigating exosomal miRNA before and after PE is a feasible approach that can be employed in the identification of a molecular signature for potential prediction of treatment response. Systems biology approaches integrating various omics data is required for identifying definitive molecular patterns associated with the disease. These studies performed on large number of samples available through biobanks from multiple sites will be needed to determine both markers associated with the diseases and for elucidation of biological pathways and processes altered under disease states [34].

## Additional files

**Additional file 1: Table S1.** Raw data for expression of serum exosomal miRNAs in CRPS patients. Raw data and relative gene expression analysis following Taqman low density array for the estimation of 754 miRNAs in the serum exosomes of CRPS patients before and after PE.

**Additional file 2: Table S2.** Plasma levels of cytokines in CRPS patients following plasma exchange (PE). Plasma levels of Fractalkine (0.33), IFNγ (0.47), IL-10 (0.51), MIP-3a (0.79), IL-17A (0.31), IL-1β, IL-2 (0.18), IL-4 (1.07), IL-6 (0.11), IL-8 (0.12),MIP-1β (0.69), TNFα (0.16), IL-13 (0.24), and IL-23 (3.06) were determined using MILLIPLEX MAP Human High Sensitivity T Cell Panel. Included in brackets next to each analyte is the sensitivity in pg/mL of the assay used to determine its plasma level.

## Abbreviations

ANOVA: analysis of variance
BMI: body mass index
CRPS: complex regional pain syndrome
CT: cycle threshold
IL-6: interleukin 6
IVIG: intravenous immunoglobulin
LPS: lipopolysaccharide
Na: non-responders after PE
Nb: non-responders before PE
NRS: numerical rating scale
PE: plasma exchange
Ra: responders after PE
Rb: responders before PE
TEM: transmission electron microscope
UTR: untranslated region
